# An Interpretability Method for Broken Wire Detection

**DOI:** 10.3390/s25134002

**Published:** 2025-06-27

**Authors:** Hailong Wu, Shaoqing Liu, Zhanghou Xu, Zhenshan Ji, Mengpeng Qian, Xiaolin Yuan, Yong Wang

**Affiliations:** 1 School of Computer Science and Engineering, Anhui University of Science and Technology, Huainan 232001, China; 2023201838@aust.edu.cn; 2 Hefei Institute of Physical Sciences, Chinese Academy of Sciences, Hefei 230031, China; 3 Institute of Energy, Hefei Comprehensive National Science Center (Anhui Energy Laboratory), Hefei 230031, China; liushaoqing@ie.ah.cn

**Keywords:** object detection, interpretability AI, broken wire detection, electromagnetic signal, LIME, RISE, D-RISE

## Abstract

As an indispensable piece of production equipment in the industrial field, wire rope is directly related to personnel safety and the normal operation of equipment. Therefore, it is necessary to perform broken wire detection. Deep learning has powerful feature-learning capabilities and is characterized by high accuracy and efficiency, and the YOLOv8 object detection model has been adopted to detect wire breaks in electromagnetic signal images of wire rope, achieving better results. Nevertheless, the black box problem of the model brings a new trust challenge, and it is difficult to determine the correctness of the model’s decision and whether it has any potential problems, so an interpretability study needed to be carried out. In this work, a perturbation-based interpretability method—ESTC (Eliminating Splicing and Truncation Compensation)—is proposed, which distinguishes itself from other methods of the same type by targeting the signaling object instead of the ordinary object. ESTC is compared with other model-agnostic interpretable methods, LIME, RISE, and D-RISE, using the same model on the same test set. The results indicate that our proposed method is objectively superior to the others, and the interpretability analysis shows that the model predicts in a way that is consistent with the priori knowledge of the manual rope inspection. This not only increases the credibility of using the object detection model for broken wire detection but also has important implications for the practical application of using object detection model to detect wire breaks.

## 1. Introduction

**Figure 1:**
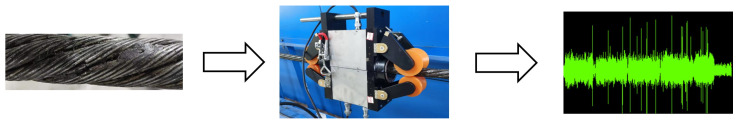
The process of manual rope inspection.

Wire rope is extensively used in the industrial field due to its high strength, excellent toughness, and wear resistance, and it has become an indispensable and important material in the industrial field. Affected by factors such as high strength, long-time load, and environment, steel wire rope is susceptible to problems such as broken wire, wear, corrosion, and deformation. To ensure the safety of personnel and the stable operation of equipment, it needs to replace steel wire rope, the breaking force of which does not meet the requirements of the current regulations, to avoid unnecessary losses caused by fracture accidents. Consequently, it is increasingly important to detect the wire rope. An important index to determine whether the wire rope needs to be replaced is whether there is a broken wire. When the number of broken wires in a lay pitch exceeds a certain number, it should be replaced. The manual rope inspection process is presented in Figure 1. The principle of the detection device is the electromagnetic magnetic flux leakage detection method [1,2], which has the characteristics of non-contact, strong anti-interference, high accuracy, and superior efficiency. The original signal is obtained by putting the wire rope under sensor detection of the detection equipment; after signal processing, the signal is converted into a signal image, and then the broken wire is marked by manual observation.

With the rapid advancement of deep learning, the IoT field is becoming more closely integrated with deep learning technologies [3], and manual rope inspection has become inefficient and difficult to integrate into automation. Compared with deep learning, machine learning methods cannot efficiently deal with the complex and diverse conditions of wire rope breakage and are not as accurate as manual rope inspection. Considering this, it is proposed to transform wire rope breakage signals into image data non-destructively through visualization and then adopt object detection to learn the wire rope breakage signals in a data-driven way. In this approach, the process of manual rope inspection can be simulated while reflecting the powerful feature extraction capability of deep learning and its strong adaptability to complex scenes, leading to accurate and efficient wire rope breakage detection. The hardware adaptability and portability of the target detection algorithm make it easier to integrate this approach into automation systems.

Existing object detection methods can be divided into one-stage and two-stage detectors according to whether the candidate region is produced or not. Two-stage detectors, such as the RCNN series [4,5,6,7], RFCN [8], SPPNET [9], etc., have two stages of explicitly generating the candidate region and performing object classification and localization. They have higher accuracy but slower speed and are suitable for scenes requiring higher detection accuracy. One-stage detectors, such as the YOLO series [10], SSD [11], etc., combine candidate region generation and object classification and localization in one stage, directly predicting the target location and accumulating chants from input images. They have faster speed and slightly lower accuracy and are suitable for real-time detection and embedded devices and other scenes requiring higher speed. In the semantic segmentation task, the DeepLab series [12,13] developed by the Google team is good at dealing with complex scenes with multi-scale context information. Song et al. [14] used a DeepLab v3 convolutional neural network architecture and ResNet-50 [15] as the backbone network for binary image segmentation to achieve pixel-level cancer detection. EfficientNet [16] is a general deep learning model architecture. Its design concept focuses on efficient feature extraction, breaking the bottleneck dictating that the accuracy and efficiency of traditional models cannot be combined, and it can be widely adapted to a variety of computer vision tasks. Kabir et al. [17] realized the triple optimization of accuracy, efficiency, and cost through EfficientNet, providing a universal automation solution for durability evaluation of building materials.

Though object detection achieves good performance, the complex structure of the model, the large number of parameters, the abstract feature representation, etc., not only bring the black box problem of deep learning [18,19,20,21], leading to poor interpretability and difficulty in performing fault detection and optimization, but also bring a crisis of confidence. Particularly, the condition of the wire rope directly affects the smooth production and the safety of personnel’s lives and properties; thus, it is necessary to understand the basis of the model for decision-making. To overcome the challenges posed by the black box problem of deep learning, deep learning interpretability plays a crucial role.

Deep learning interpretability is the key to improving model understanding and trust, discovering model flaws, and guiding model adjustments. According to the principle of explaining the basis of model decision-making, interpretability methods can be divided into two types—gradient-based methods and perturbation-based methods—both of which mainly visualize the regions that are important for model decision-making using a saliency map. The principle of the gradient-based interpretability method is to quantify the importance of input features for model prediction by calculating and analyzing the gradient of the model output on the input features. Zhou et al. [22] developed a visualization technique called CAM, which generates class activation maps for interpretable analysis using the output feature maps of the global average pooling layer. Selvaraju et al. [23] extended CAM and devised Grad-CAM to use it with a broader range of modeling architectures. Chattopadhay et al. [24] presented Grad-CAM++, which generates more accurate class activation maps by further optimizing Grad-CAM. Wang et al. [25] designed Score-CAM, which generates class activation maps by recalculating the feature scores after masking. Layer-CAM [26] generates more comprehensive and high-resolution class activation maps by combining feature maps from several convolutional layers. Perturbation-based interpretability methods are model-agnostic, as they generate saliency maps by perturbing input features or regions and then observing changes in the model outputs, finally evaluating the features or regions that have a large impact on the model. Zeiler and Fergus [27] aimed to understand the behavior of a model through occlusion analysis. Ribeiro et al. [28] developed LIME, a black box interpretation method for locally interpretable models. This method approximates the predictive behavior of a complex model with a simple and interpretable model as a local neighbor of the samples to be interpreted. The core idea of RISE [29] is to generate a random mask obscuring the image region and generate a saliency map with a weighted average of the masks, making it mainly applicable to image classification models. Compared with RISE, D-RISE [30] has improved object detection, and its application scope is more expansive. Certainly, there are some problems in the gradient-based and perturbation-based methods. To resolve these problems, Chen et al. [31] put forward a visually accurate search method, which generates accurate attribution maps with fewer regions.

The existing classical perturbation-based interpretability methods are aimed at ordinary targets rather than signal targets. Signal targets often have physical constraints, have stronger integrity and continuity compared to ordinary targets, and are closely related to each feature. RISE and D-RISE interfere with the disturbance mode of input data by generating random occlusion modes. This method cannot guarantee the continuity and integrity of the signal context in the process of disturbing the electromagnetic signal of the wire rope. At the same time, the disturbance-based method does not consider the distortion problem caused by signal truncation during the disturbance process, which leads to inaccurate feature attribution. In order to solve the above problems, this paper considers electromagnetic signals of wire ropes and proposes a model-agnostic perturbation-based post hoc localizable interpretability method called ESTC (Eliminating Splicing and Truncation Compensation). This method uses compensation and splicing to avoid the problem of signal distortion and destruction of signal continuity after truncation. At the same time, interpretability analysis is performed for the characteristics of different regions of the signal. The main contributions of this work are as follows:

- The electromagnetic signal of the wire rope is transformed into image data and made into a dataset through manual labeling. Then, the dataset is divided into a training set, a validation set, and a test set to train the YOLOv8 [32] object detection model for broken wire prediction.
- A new post hoc locally interpretable perturbation-based method called ETSC is proposed. ETSC distinguishes itself from other perturbation-based methods in the perturbation mode as it adopts corresponding perturbation modes according to the characteristics of different regions of the broken wire electromagnetic signals, specifically using the elimination of splicing for the middle region of the broken wire signals and using the truncation of the compensation for the two-end region. The perturbation method does not cause damage to the signal and avoids a significant reduction in the confidence score of the model due to the failure to recognize the signal characteristics.
- By using the ETSC method of perturbation, the impact of the weight on the model in different regions of the broken wire signals can be derived. The saliency map produced using the weight can intuitively show the influence of the model decision-making degree in different regions of the broken wire signals. Then, the model decision-making is understood based on the broken wire signals of the main characteristics of the method, which is important to promote the practical application of the method of deep learning.
- The proposed interpretable method ETSC is compared with three model-agnostic perturbation-based interpretable methods, LIME, RISE, and D-RISE. The results indicate that the saliency map obtained by ETSC can obtain accurate and reliable results, demonstrating that the decision-making behaviors of the model are consistent with existing methods of artificial rope inspection.

## 2. Materials and Methods

### 2.1. Wire Rope Dataset Production and Processing

**Figure 2:**
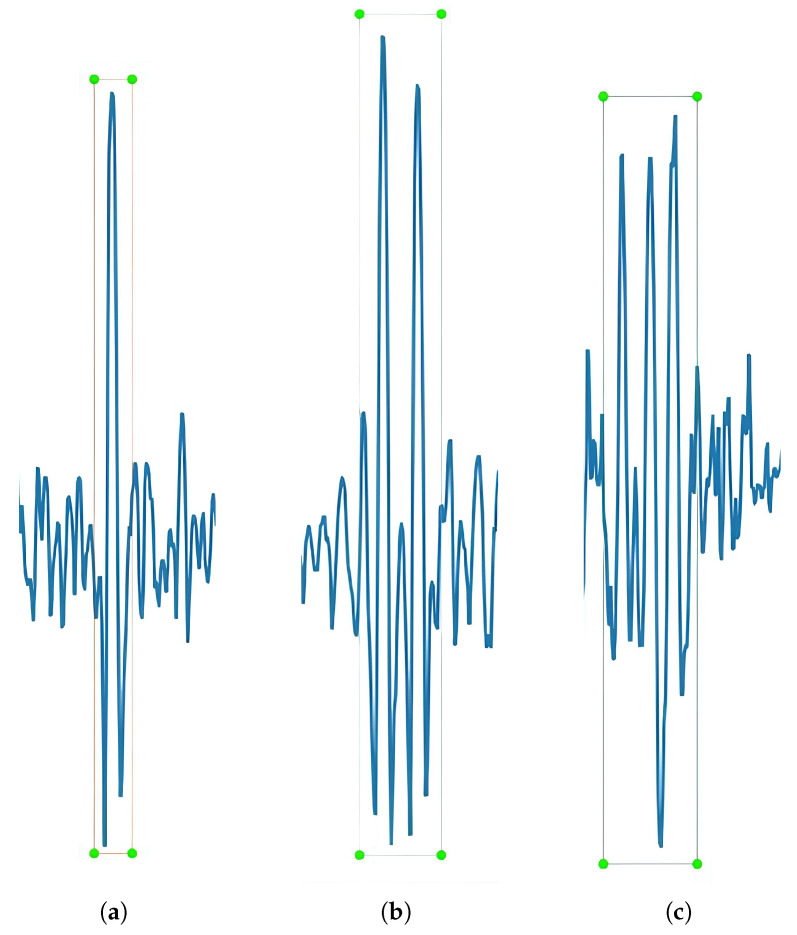
Three categories of broken wire signals in most cases, and the number of troughs is twice the number of peaks: (**a**) single broken wire, (**b**) double broken wire, and (**c**) triple broken wire.

Some of the wire ropes used in different mines were selected, and broken wire detection devices were employed to collect electromagnetic signals from each wire rope, where the sensors detected electromagnetic signals containing location information and defect information. This information was transmitted to different information sites through shielded cables. Then, these signals were further processed (e.g., signal denoising) at these sites, and the numerical data of each wire rope was finally obtained. Some software was used to plot the data of each wire rope into a certain number of images, and then LabelImg was utilized to label broken wires on the images produced for these different wire ropes. Only three target categories were set: single broken wire, two broken wires, and three broken wires. The three signal categories are not the same, and the larger the category, the smaller the number. Even though there may be more types of broken wire, the data is too sparse to be set, and the different types of broken wire are illustrated in Figure 2. After filtering out some invalid images caused by the vibration of the wire rope during the acquisition process, despite the limited number of broken wire ropes, more than 3500 broken wire images were collected for the broken wire dataset. The collected images were used for training and validation at a ratio of 8:2, and then the 600 signal images were used for the test set alone; these images are independent of the training and validation sets, preventing evaluation bias due to data leakage. The test set comprehensively covers all broken wire types and various wire rope types encountered during training, ensuring dataset diversity.

The dataset includes three categories of broken wire conditions for different types of steel wire ropes, where “different types” denote ropes from distinct mining regions. These rope types differ in electromagnetic signal characteristics. Moreover, the dataset shows substantial class imbalance among the three classes: single broken wires, double broken wires, and triple broken wires. The number of single broken wires significantly exceeds that of double and triple ones at a ratio of approximately 8.5:1:0.5. In practice, the number of double and triple broken wires is likely even lower, a phenomenon common in operational steel wire ropes. A higher number of multi-broken wires typically indicates the rope has approached or met the scrap standard. This imbalance causes the YOLOv8 model to demonstrate poorer fitting performance for multi-broken wires than single ones. To mitigate this, data augmentation was applied to multi-broken wire samples to improve the model’s generalization capability.

### 2.2. YOLOv8 Model Experimental Process and Environment

Considering the possibility of deploying the model in edge devices to integrate it into automation systems, the size of the selected model parameters and the required computational resources should not be too large so as not to exceed the storage and processing capacity of the edge device. Meanwhile, the detection speed cannot be too slow; otherwise, it will affect the real-time response performance of the whole automation system, thereby reducing the operational efficiency and reliability.

To determine the performance gap between two-stage and one-stage object detection algorithms, a representative two-stage algorithm, Faster R-CNN, and a representative one-stage algorithm, YOLOv8, were selected. We compared Faster R-CNN and YOLOv8 on the same dataset and in the same hardware conditions(Nvidia RTX4060); the results are shown in Table 1. It was found that Faster R-CNN has much larger model parameters and higher inference memory than YOLOv8. Moreover, its FPS is lower than YOLOv8, and it fails to reach the real-time detection standard, while its mAP also lags behind YOLOv8. Therefore, we ceased selecting two-stage detection algorithms. Notably, the YOLO series describes a group of end-to-end one-stage object detection algorithms, which have the advantages of fast detection speed, good real-time performance, strong generalization ability, easy deployment, and iterative updating. So, we chose one of the most stable and reliable versions, YOLOv8, for experiments and interpretability analysis.

**Figure 3:**
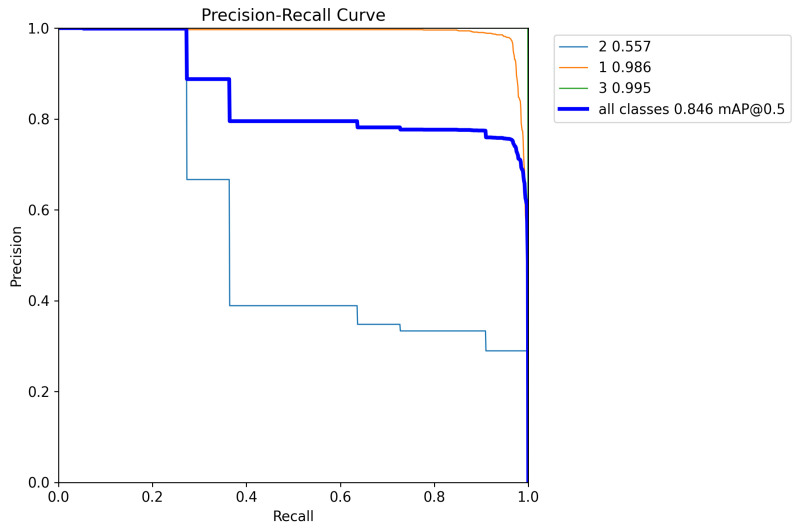
The PR curves for YOLOv8 model.

The model was trained on eight Nvidia RTX3090 GPUs. The model structure of YOLOv8 was not modified, and only the training parameters were modified to obtain the best weight file—specifically, the number of epochs was set to 500, batch size was set to 64, and the number of warmup epochs was set to 10. These parameters were determined through an exhaustive search conducted using a fixed dataset, fixed hardware configurations, and fixed hyperparameters. The optimal combination was validated using mAP, and we performed a loss comparison between the training and validation sets and considered test set metrics including accuracy and false detection rate. The reason for why we did not modify the structure of YOLOv8 is because interpretability analysis needed to be conducted on the model afterward; interpretability analysis can be easily used to discover potential problems in the model or dataset. The final PR curve was obtained and is shown in Figure 3.

### 2.3. Defects of the Traditional Perturbation Method

#### 2.3.1. RISE Methodology Modification

Since the RISE method is mainly used to model image classification and YOLOv8 belongs to the object detection model, it is impossible to directly apply the RISE method to YOLOv8. To this end, this paper has made the following two modifications to RISE to make it applicable to YOLOv8 for interpretability analysis:

- The image preprocessing method in RISE differs from that in the YOLOv8 model. To use the YOLOv8 model for inference, the image preprocessing method in RISE needs to be modified to align with that in YOLOv8;
- In the RISE interpretable method, before multiplying the processing masks and weight scores, the weight scores are acquired by the image classification model after softmax processing, while the results acquired by the YOLOv8 model after inference are different. In this paper, the confidence scores of the target categories in the image were obtained from the YOLOv8 model after inference with non-maximum suppression, which was multiplied with the produced random mask to acquire the saliency map.

#### 2.3.2. Inappropriate Perturbation Method

**Figure 4:**
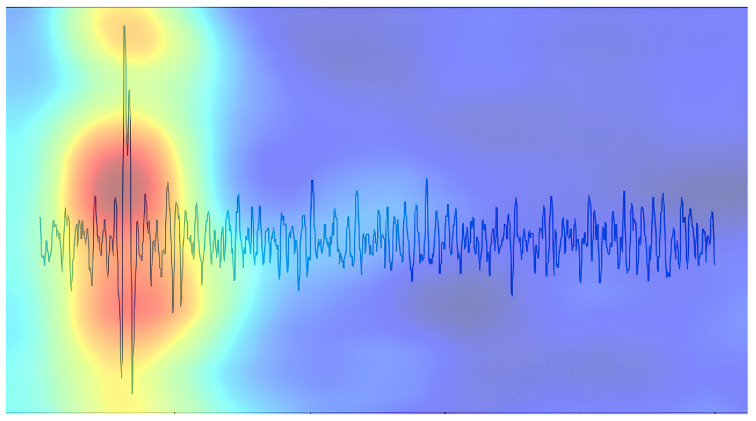
Results of the RISE method applied on the YOLOv8 model.

After applying the RISE method to the YOLOv8 model, it was found that the highlighted red area in the saliency map is always concentrated in the middle of the broken wire signal, as illustrated in Figure 4. Though manual rope testing is performed based on the change in the amplitude of the broken wire signal and the characteristics of the signal peaks and troughs, the results obtained by the RISE interpretability method are opposite to manual rope testing.

To determine whether the reason for this result is that the model mainly focuses on the middle region of the broken wire signal for discrimination or whether there is a problem with this perturbation method, experiments were designed.

**Figure 5:**
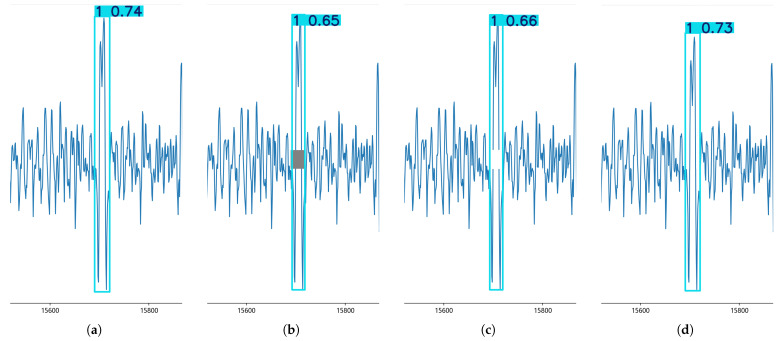
Comparison of two different perturbation approaches. (**a**) The results after the YOLOv8 model predictions have a confidence score of 0.74. (**b**) After masking the middle region of this signal using a gray pixel block, the confidence score is reduced to 0.65, as predicted by the YOLOv8 model. (**c**) Adjusting the pixel value of the gray pixel block to a white pixel block consistent with the background leads to a confidence score of 0.66, as predicted by the model. (**d**) Splicing the upper and lower regions of the interrupted (**c**) broken wire signal leads to a confidence score of 0.73, as predicted by the YOLOv8 model.

Note that the RISE interpretability method utilizes random masks for perturbation in a similar way to region masking, both of which work by eliminating pixels from the image. Then, two perturbation approaches were designed in the broken wire signal, i.e., masking the pixel blocks and eliminating the masked areas and splicing the signal, to determine the efficacy of this masking approach by comparing the confidence scores derived from the YOLOv8 model predictions, and the results are presented in Figure 5.

Both methods illustrate the impact of the same perturbed region on the model prediction, but the masking approach destroys the signal’s continuity and integrity, characteristics of the signal, and it introduces new features of the pixel block, causing the model prediction confidence score to decrease significantly.

In contrast, the use of splicing removal not only eliminates the influence of regions on the model but also ensures the continuity and integrity of the broken wire signal without introducing new features. This shows the inappropriateness of using perturbations such as region masking on the broken wire signal and also suggests that the model has a focus on the middle region of the broken wire signal, but this is not significant.

### 2.4. A New Way of Perturbing

**Figure 6:**
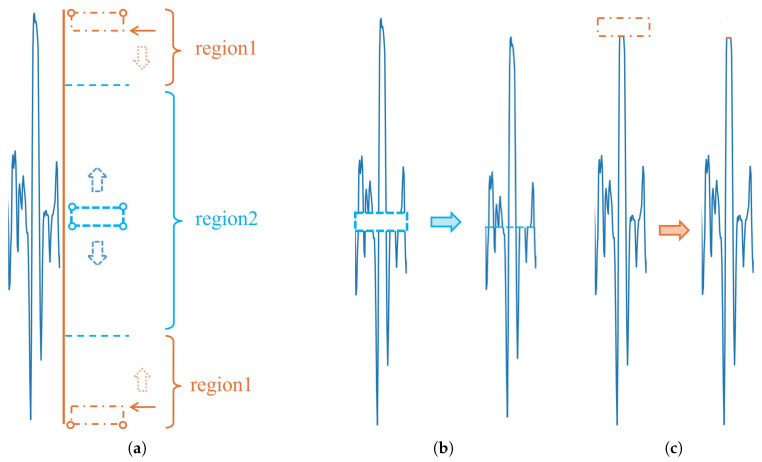
Different perturbation methods are used for different regions of the broken wire signal. (**a**) In region 1, one end of the truncation box is fixed, and its height varies with the number of perturbations until it covers the boundary region. In region 2, the elimination box has a fixed size, and it will move in region 2 until it covers the entire intermediate region. (**b**) The eliminating and splicing process. (**c**) The truncation compensation process.

Two different perturbation methods are used for the broken wire signal in the middle region and the two ends of the region with different characteristics, respectively. Elimination splicing in the middle region and truncation compensation at the two ends of the region are both perturbation methods used to ensure the continuity and integrity of the signal, as shown in Figure 6.

Initially, the position of the bounding box and the confidence score of the image broken wire signal are acquired through model inference. The diagonal coordinates of the detection box are specified as $\left(x_{1},y_{1}\right),\left(x_{2},y_{2}\right)$, respectively. The corresponding confidence score is represented as P. The ratio of the range of the center region of the bounding box in the vertical direction to the regions at the two ends is 6:4. This ratio is derived from experiments and is optimal. The methods of calculating the height, width, and height of the central area of the fault signal are given by

(1) $$H_{\mathrm{signal}\;}=\left|y_{2}-y_{1}\right|$$

(2) $$W_{\mathrm{signal}\;}=\left|x_{2}-x_{1}\right|$$

(3) $$H_{\mathrm{center}}=\frac{3\times H_{\mathrm{signal}}}{5}$$

In the eliminating and splicing perturbation method, the elimination box is set to have the same width as the bounding box. The central region of the broken wire signal exhibits stable and smooth characteristics, whereas the terminal regions display sharp fluctuations and pronounced variability, necessitating separate processing for these two segments. The central region constitutes the majority of the broken wire signal, while the terminal regions comprise a smaller proportion. After testing ratios of 7:3 and 8:2, it was observed that these proportions inadequately encompass the terminal regions. Conversely, a 6:4 ratio effectively covers both terminal regions without excessively encroaching upon the central segment.

During the experiment, it was observed that when the height of an elimination box exceeds 1/24th the height of the central region, discontinuities occur at the splice points, causing signal misalignment across the junction. Therefore, the height of the elimination box must be constrained, and it is necessary to ensure that the height of the central region is an integer multiple of the elimination box height. This ensures the elimination box moves in integer steps, allowing its coverage to precisely span the entire central region. Thus, the elimination box height should be set to, at most, 1/24th that of the central region. The width, height, and movement range of the elimination box are given by

(4) $$W_{\mathrm{elimination}\;\mathrm{box}}=W_{\mathrm{signal}}$$

(5) $$H_{\mathrm{elimination}\;\mathrm{box}}=\frac{H_{\mathrm{center}}}{n}\;\left(n\geq 24,n\in N^{*}\right)$$

(6) $$y_{1}+\frac{H_{\mathrm{signal}}}{5}\leq y\leq y_{2}-\frac{H_{\mathrm{signal}}}{5}$$

Let the diagonal coordinates of the bounding box of the fault signal after each eliminating and splicing perturbation be $\left(x_{3},y_{3}\right),\left(x_{4},y_{4}\right)$. Then, after this perturbation, the coordinates of the bounding box of the fault signal are calculated as follows:

(7) $$x_{3}=x_{1},x_{4}=x_{2}$$

(8) $$\left\{\begin{array}{cc} y_{3}=y_{1}+H_{\mathrm{elimination}\;\mathrm{box}},y_{4}=y_{2}, & y_{1}+\frac{H_{\mathrm{signal}}}{5}\leq y\leq y_{1}+\frac{H_{\mathrm{signal}}}{2}\\ y_{3}=y_{1},y_{4}=y_{2}-H_{\mathrm{elimination}\;\mathrm{box}}, & y_{1}+\frac{H_{\mathrm{signal}}}{2}\leq y\leq y_{2}-\frac{H_{\mathrm{signal}}}{5} \end{array}\right.$$

Considering that the height of the center region of the original bounding box is n times that of the elimination box, this eliminating and splicing perturbation is executed n times. Let the confidence score of the model prediction after each eliminating and splicing perturbation be $P_{\mathrm{eliminate}}^{i}\left(i\leq n\right)$, and the difference is normalized; then, the weight of this perturbed region after executing each elimination perturbation is as follows:

(9) $$w_{i}=\frac{\left|P-P_{\mathrm{eliminate}}^{i}\right|}{P}\;\left(i\leq n\right)$$

For the perturbation method of truncation compensation, the truncation box is fixed at one end in the vertical direction, and after each perturbation, the height of the truncation box is doubled up to the boundary of the center region. The compensation is used to keep the truncated signal continuous. Specifically, pairs of truncation points are observed at the truncation location and then connected using straight lines of the same pixel as the signal. The calculation of the width and height of the truncation box are given by

(10) $$W_{\mathrm{truncation}\;\mathrm{box}}=W_{\mathrm{signal}}$$

(11) $$H_{\mathrm{truncation}\;\mathrm{box}}=\frac{H_{\mathrm{signal}}}{k}\;\left(k\geq 30,k\in N^{*}\right)$$

Let variable i be the number of perturbations; after each truncation compensation perturbation, the height of the truncation box varies as follows:

(12) $$H_{\mathrm{truncation}\;\mathrm{box}}^{i}=H_{\mathrm{truncation}\;\mathrm{box}}\times i\left(\frac{i}{k}<\frac{1}{5},i\in 1,2,3\ldots \right)$$

Let the diagonal coordinates of the bounding box after the truncation and compensation of the fault signal be $\left(x_{5},y_{5}\right),\left(x_{6},y_{6}\right)$; then, its coordinates are calculated as follows:

(13) $$x_{5}=x_{1},x_{6}=x_{2}$$

(14) $$\left\{\begin{array}{cc} y_{5}=y_{1}+H_{\mathrm{truncation}\;\mathrm{box}}^{i},y_{6}=y_{2}, & y_{1}\leq y_{i}\leq y_{1}+\frac{H_{\mathrm{signal}}}{5}\\ y_{5}=y_{1},y_{6}=y_{2}-H_{\mathrm{truncation}\;\mathrm{box}}^{i}, & y_{2}-\frac{H_{\mathrm{signal}}}{5}\leq y_{i}\leq y_{2} \end{array}\right.$$

Denote the confidence score of the output predicted by the model after each truncation–compensation perturbation as $P_{\mathrm{truncation}\;\mathrm{box}}^{j}$ and the total number of perturbations at both ends of this fault signal as j. Then, the corresponding weights after each perturbation are calculated as follows:

(15) $$w_{j}=\frac{\left|P-P_{\mathrm{truncation}\;\mathrm{box}}^{j}\right|}{P}$$

The perturbation weights consisting of $w_{i}$ and $w_{j}$ are the weights of the impact of each region of the fault signal in an image on the model. Even if the image contains many fault signals, it is possible to use this method to calculate the weights of different regions of all the fault signals affecting the model.

Based on the weights obtained for each perturbed region in the detection box, the weight value can be mapped to a pixel range of 0–255 using different pixel values rather than weights for visualization. Then, a separate saliency map of the pixel values produced by the weights in each detection box in the image is drawn, and Gaussian blurring is utilized to remove sharp edge regions. Since a saliency map of the original image size is generated for each detection box in the figure, multiple detection boxes yield multiple individual saliency maps. Overlaying these on the original image may compromise clarity. To address this, all non-zero pixels from the generated saliency maps are integrated into a single-composite saliency map based on their spatial positions, which is then overlaid onto the original image. This approach avoids multiple overlays that could diminish image clarity.

### 2.5. General Overview of ESTC Application on YOLOv8

**Figure 7:**
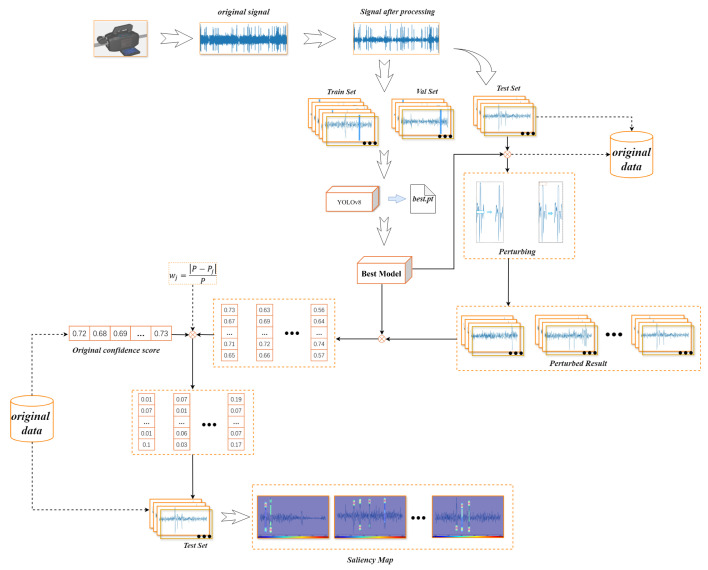
Overview of ESTC application on YOLOv8.

Finally, the ESTC method was applied to the YOLOv8 model, and its processing is illustrated in Figure 7. First, a broken wire signal dataset was constructed by manual labeling after signal processing (wavelet transform, etc.) of the signals acquired from the sensor acquisition, where the training set and validation set were used for model training to obtain the best model. Then, the best model was utilized for test set prediction to obtain the confidence score and location information of each broken wire signal on the test set. Taking an image on the test set as an example, if there is a broken wire signal in the image, a batch of perturbed images will be produced after the perturbation, with each image representing a perturbed region of the broken wire signal, and the batch of images will cover the entire broken wire region. Then, through the inference of the YOLOv8 model, the confidence score of each region of the broken wire signal after perturbation will be obtained. The difference between the unperturbed confidence score and the perturbed confidence score is normalized. The weights of the different regions of the image that affect the model are finally obtained, and a saliency map is acquired by overlaying them on the original image.

## 3. Results and Discussion

This paper focuses only on the interpretability analysis of visualizations. To ensure the scientific validity and accuracy of the comparisons, D-RISE, RISE, LIME, and our proposed ESTC method were selected for comparison on the same test set using the same YOLOv8 model weight file. According to the priori knowledge accumulated from manual rope testing, broken wires are mainly judged based on the amplitude of the signal, as well as the shape of the peaks and troughs at the two ends and other features. It is noteworthy that all the interpretable methods are model-agnostic, fully demonstrating that they are sufficiently objective.

Firstly, the highlighted red areas in the saliency map are represented as heat values and are considered as the basis for modeling decisions. Then, the model decision basis produced by each interpretability method is compared with the manual decision basis. If the model decision basis produced by a certain interpretability method better fits with the manual decision basis, it suggests that the interpretability method is superior, indicating that it is more suitable for interpretable analysis of model detection of broken wire signals. The following will show the performance of these four methods on different wire rope categories and multi-target images.

**Figure 8:**
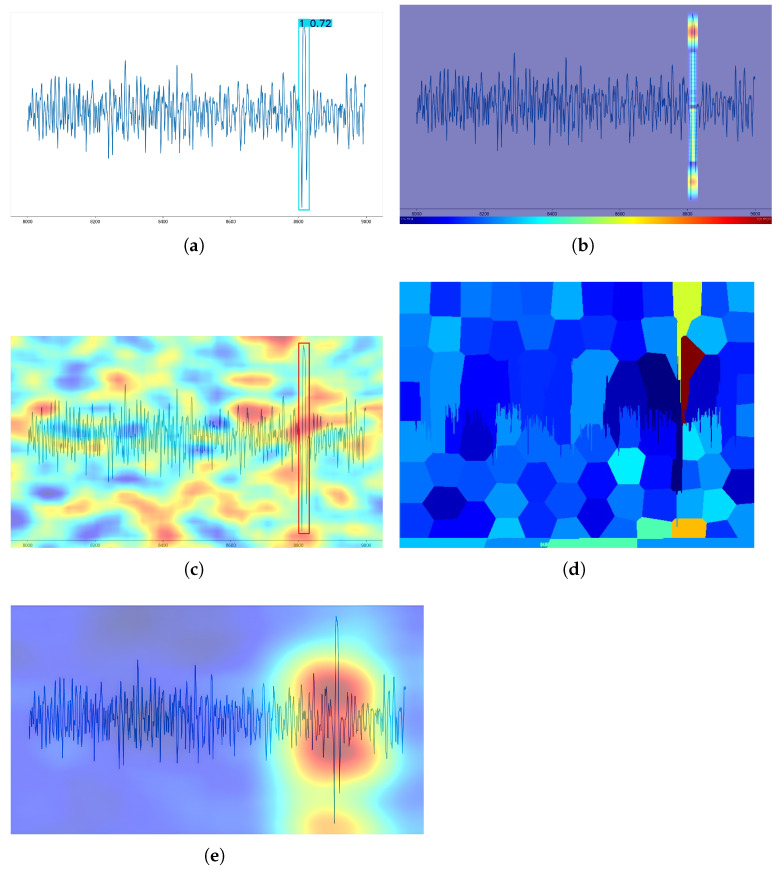
Four interpretability methods were employed to analyze the images with broken wire signals classified into the single-broken-wire category. (**a**) The original image predicted by the YOLOv8 model to belong to the category of single broken wires with a confidence score of 0.72. (**b**) Interpretability analysis results for ESTC with n-ratio set to 1/30 (elimination box size ratio) and k-ratio set to 1/50 (truncation box size ratio). (**c**) Interpretability analysis results for D-RISE. (**d**) Interpretability analysis results for LIME, where the number *num-samples* of samples produced by LIME is 3000. (**e**) Interpretability analysis results for RISE, where the number *N* of masks in RISE is 6000, the grid size *s* is 8, and the sparsity ratio *p*1 of masks is 0.2.

Figure 8 illustrates the results of applying four different interpretable methods to the YOLOv8 model in an image with only one target, and the category is the single-broken-wire category. Regarding the accuracy of the location of the produced heat values, ESTC and RISE all produced heat values at the location of the broken wire signal, except for D-RISE, while LIME also produced a block of pixels in dark red at that location. While D-RISE’s heat distribution is around the image and fails to determine how the model generates a decision basis, LIME cannot locate the exact area of the broken wire signal due to the large pixel block. The heat distribution produced by RISE is concentrated in the middle region of the broken wire signal and covers nearly the entire broken wire signal, which only demonstrates that the model pays attention to the entire broken wire signal, especially the middle region, due to the large range of heat values. By contrast, for the ESTC method proposed in this paper, its heat distribution is mainly concentrated in peaks and troughs of the broken wire signal, fitting the best with the manual decision-making basis, indicating that the ESTC method performs better than the other methods in the single-target category of the broken wire image.

**Figure 9:**
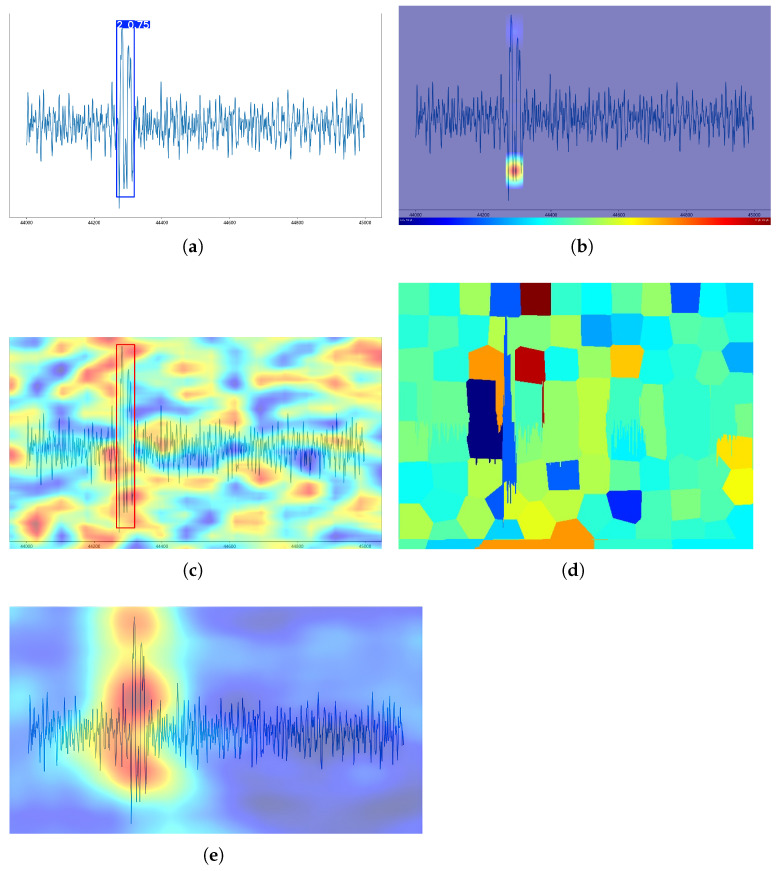
Four interpretability methods were used to analyze the images that were classified as double-broken-wire signals. The parameters of all methods are the same as those in Figure 8. (**a**) The original image predicted by the YOLOv8 model to belong to the category of single broken wires with a confidence score of 0.75. (**b**) Interpretability analysis results for ESTC (**c**) Interpretability analysis results for D-RISE. (**d**) Interpretability analysis results for LIME. (**e**) Interpretability analysis results for RISE.

Figure 9 shows the interpretability analysis of the four interpretability methods against the YOLOv8 model in the double-broken-wire category of the wire rope break image. The saliency map presented by the ESTC method indicates that the heat values are concentrated at the trough position of the broken wire signal. The D-RISE method employs a detection box to label the target, but the heat distribution is not only around the target but also in the blank area. The LIME method exhibits dark pixel blocks in the target region to differentiate them from the other light-colored pixel blocks, suggesting that it has identified the target region. The RISE method nearly covers the whole target area.

To summarize the comparison, it can be observed that the heat map presented by the D-RISE method does not provide effective assistance for the model to perform interpretable analysis, and the LIME method can locate the more important pixel block areas in the target region, but due to its large pixel block region, it can only conduct a coarse-grained analysis. Though the RISE method provides almost full heat coverage of the entire target region, it is not conducive to analyzing the impact of different regions of the broken wire signal on the model. The method presented by ESTC can reveal the influence of different regions of the target on the model, indicating that the trough location is among the more important regions relative to the other locations of the broken wire signal of the type, while providing a finer granularity of of interpretation.

**Figure 10:**
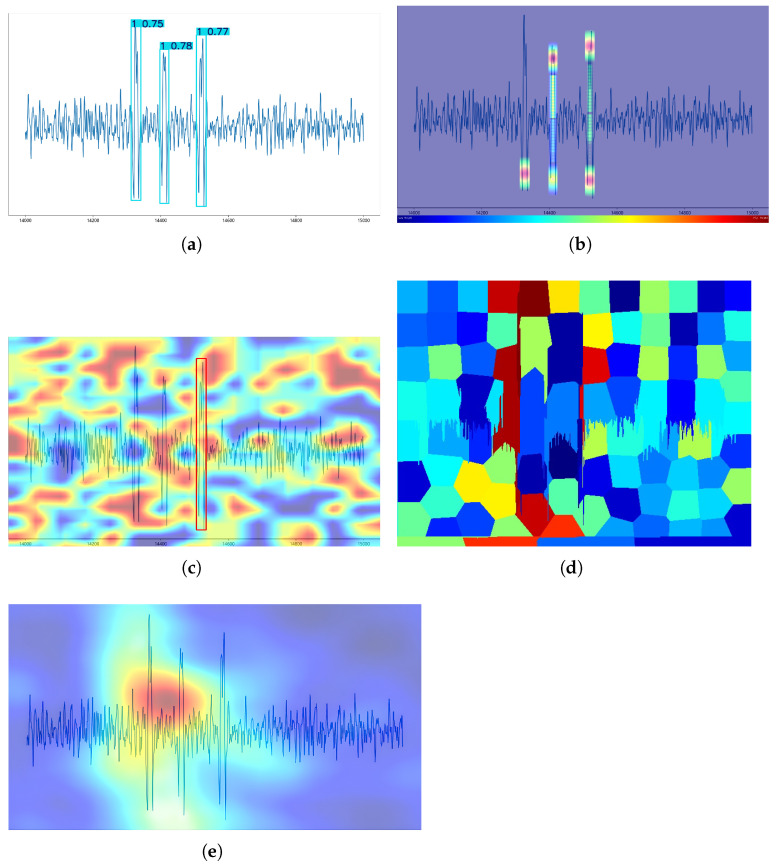
Four interpretability techniques were used to conduct a comprehensive analysis of the images categorized as multi-broken wire signals. The parameters of all methods are the same as those in Figure 8. (**a**) The original image predicted by the YOLOv8 model to belong to the category of single broken wires with a confidence score of 0.75. (**b**) Interpretability analysis results for ESTC. (**c**) Interpretability analysis results for D-RISE. (**d**) Interpretability analysis results for LIME. (**e**) Interpretability analysis results for RISE.

Figure 10 shows the interpretability analysis results of the model when there are multiple targets in the broken wire signal image. In the saliency map presented by ESTC, there is heat distribution on each target, concentrated at the two ends of the target, and it should be noted that the heat in the left target is mainly concentrated at the lower end. The detection box in D-RISE only labels a single object, while the heat is distributed in all regions of the image, so it is impossible to determine which regions have a large impact on the model. The LIME method generates blocks of red pixels distributed across many target regions, with each pixel block covering a wider area. The heat produced by RISE does not cover all the targets but is concentrated in the upper-middle region of the two targets on the left side, while the heat distribution of the targets on the right side is not evident.

The comparison results indicate that ESTC outperforms the other three methods, attributed to its more fine-grained explanatory strength, heat distribution for all targets, and the generation of a model decision basis most compatible with manual decision-making.

The above four interpretability methods were applied to the entire test set, extensive comparative analyses were carried out, and the performance results were all identical to those of the three experiments described above, where the images in the three comparative experiments were randomly selected from each category in the test set.

When YOLOv8 detects wire rope defects, false positives may occur (—for example, misclassifying normal signals as broken wire signals). Statistical analysis of the test set shows that when background noise increases signal amplitude globally, false detection rates rise because broken wire signals have amplitudes similar to normal ones. This phenomenon is likely caused by wire rope vibrations during sensor data collection. As an interpretable perturbation-based method, ESTC is model-agnostic, meaning its saliency maps can only reflect the contribution weights of signal components during correct or incorrect model detections.

Currently, the ESTC methodology has been validated only for wire rope electromagnetic signal detection. The employed perturbation strategy relies on significant feature differences between target and noise signals, for example, in ECG, where target signals are easily distinguishable from noise signals, which indicates potential applicability. However, for other signal types, if their time-frequency distributions and statistical characteristics differ markedly from those of wire rope signals, direct application of the ESTC approach may be ineffective. Thus, extending ESTC to new signal domains requires adapting the perturbation strategy to specific signal features, involving parameter recalibration and algorithmic optimization, requiring significant implementation costs.

In practical deployment scenarios, the ESTC faces multiple constraints: Firstly, the limited computational capability of edge devices leads to excessive inference latency. Secondly, processing large-scale, long-sequence data significantly elevates memory consumption due to perturbation calculations, often requiring custom hardware acceleration or multi-GPU parallelization. Thirdly, the complex operational conditions of wire ropes—including oil contamination and vibrational exposure—significantly hinder the model’s capability to accurately identify wire breakage signals, thereby deteriorating the interpretability and reliability of the ESTC.

## 4. Conclusions

In this study, the YOLOv8 object detection model was adopted for broken wire detection of wire rope electromagnetic signals, and the best weight files produced by the model were used for interpretability analysis. A new perturbation-based model-agnostic interpretability method called ESTC was proposed, and this method provides a novel signal perturbation approach without destroying signal continuity and integrity compared to traditional perturbation approaches used for common targets. Also, it can determine the weights of the impact of each region where the perturbation is performed on the model decision.

Compared with other model-agnostic perturbation-based interpretability methods, our method shows a finer-grained interpretation, and its interpreted model decision behavior is the most consistent with the decision behavior of the manual rope inspection, validating its superiority for signal detection. The explanation it provides is important for improving human trust in the model for broken wire detection and for integrating the model into industrial automation systems.

With the advancement of deep learning technology, the ESTC algorithm can be integrated with more advanced deep learning models in the future. Interpretability analysis of new detection models will guide the optimization of model architectures and parameters, enabling simultaneous improvements in detection accuracy and decision interpretability. Furthermore, ESTC can be embedded into industrial IoT and intelligent diagnostic platforms, collaborating with monitoring data and diagnostic models of other industrial equipment. By interpreting the wire rope broken detection model and integrating it with operational data across the industrial system, comprehensive equipment health management and fault prediction can be achieved, providing deeper support for the intelligentization of industrial development.

## Figures and Tables

**Table 1:** Comparison of Faster R-CNN and YOLOv8n.

Model	Parameters (M)	FPS	Inference Memory (MB)	@mAP0.5
Faster R-CNN (Resnet50)	43.1 M	14.6	105.5	0.787
YOLOv8n	3.1 M	57.7	43.57	0.846

## Data Availability

The dataset and ESTC code are available at https://github.com/seadragon14/ESTC, accessed on 4 May 2025.
